# Role of Assistive Robots in the Care of Older People: Survey Study Among Medical and Nursing Students

**DOI:** 10.2196/18003

**Published:** 2020-08-12

**Authors:** Sylwia Łukasik, Sławomir Tobis, Sylwia Kropińska, Aleksandra Suwalska

**Affiliations:** 1 Institute of Human Biology and Evolution, Faculty of Biology Adam Mickiewicz University in Poznań Poznań Poland; 2 Occupational Therapy Unit, Chair of Geriatric Medicine and Gerontology Poznan University of Medical Sciences Poznań Poland; 3 Geriatrics Unit, Chair and Department of Palliative Medicine Poznan University of Medical Sciences Poznań Poland; 4 Department of Mental Health, Chair of Psychiatry Poznan University of Medical Sciences Poznań Poland

**Keywords:** assistive robots, older adults, acceptance, medical students, nursing students

## Abstract

**Background:**

Populations are aging at an alarming rate in many countries around the world. There has been not only a decrease in the number of births and an increase in the percentage of older people, but also an increase in the number of people living alone. There is growing demand for specialist medical care and daily care with the number of people who can act as caregivers reducing. The use of assistive robots can, at least partially, solve these problems.

**Objective:**

The purpose of this study was to examine the opinions of future health care professionals (medical and nursing students) regarding the use of assistive robots in the care of older people.

**Methods:**

The study was conducted with a group of 178 students from Poznan University of Medical Sciences, Poznań, Poland (110 nursing students and 68 medical students), using the Users’ Needs, Requirements, and Abilities Questionnaire.

**Results:**

The participants of this study believed that assistive robots should, first of all, remind older people to take medication regularly, ensure their safety, monitor their health status and environment, provide cognitive training, and encourage them to maintain physical activity. In the students’ opinion, the robot should not be an older person’s companion but only act as an assistant. Nursing students had significantly higher scores than medical students in several statements concerning everyday use of robots, including reminding about meals (*P*=.03), monitoring the environment (*P*=.001), providing advice about a healthy diet (*P*=.04), monitoring the intake of food and fluids (*P*=.02), and automatic “switch on” function (*P*=.02). Nursing students were more focused on the social functions of robots, including encouraging contact with friends (*P*=.003) and reducing the sense of loneliness and improving mood (*P*=.008). Medical students were more aware of privacy issues in the statement concerning the possibility of switching off the robot in specific situations (*P*=.01).

**Conclusions:**

Our study revealed a generally positive attitude of future doctors and nurses toward assistive robots, which can have an impact on their acceptance by older adults. In the future, medical professionals could help their patients to choose the right robots (and necessary functions) that are best suited to their needs. However, this would require expanding the curriculum to include the issues of gerontechnology.

## Introduction

In recent years, populations are aging at an alarming rate in many countries around the world. There has been not only a decrease in the number of births and an increase in the percentage of older people, but also an increase in the number of people living alone [1]. Europe is currently one of the oldest regions in the world [2]. According to Eurostat data, in 2014, people aged over 65 years accounted for almost 20% of the population of the European Union [3]. It is estimated that this value will increase to around 30% before 2060 [4]. The aging of the European society is now one of the biggest social and economic challenges faced by the European Union [3].

Since people are living longer and older people are constituting an increasing proportion of the population, there is progressively insufficient availability of specialized caregivers [5]. One possible form of support that has the potential to solve the problems of the aging of European societies, at least partially, is the use of assistive robots in the care of older people. Such robots can make it easier for older people to remain self-efficient and independent for longer while also reducing the burden on the family and formal caregivers [6]. Additionally, robots can not only help older adults in everyday life, but also be used in medical care (eg, for remote monitoring of patient health), which can additionally contribute to reducing costs for public services or care-assurance budgets [7].

Thus far, several models of robots supporting older people, with quite a variety of uses, have been developed. Robots can be used as aids in preparing [8] and consuming meals [9,10], daily toileting [11], doing housework [12], and monitoring the user’s state of health [2], among others. In addition, these devices can also provide older users with company (eg, as a chess companion) and encourage them to do cognitive training, as some studies have suggested the positive effects of these devices on cognitive function in older people [13]. Social robot interventions have been reported to improve mood and reduce stress levels in elderly users [14].

Advances in technology have enabled the development of robots that are closely adjusted to the needs of their users. However, this requires a thorough understanding of the needs and expectations of older people regarding these devices [15,16]. While the research conducted so far has concentrated mainly on the acceptance of existing robots [5,17,18], understanding why older people accept or reject assistive robots and what expectations they have of them is essential to not only improve the design of these robots but also develop effective strategies for placing them on the market [7].

While designing a new robot, the opinion of the end user has a central place, followed by the views of their family members and caregivers. As demonstrated by Sorri and Leinonen, the preferences of these two groups can be different [19]. Our previous study of occupational therapy students’ perceptions on the use of robots in the care of older adults indicated that students were rather skeptical regarding the abilities of older people to operate robots [20]. Furthermore, we found that older subjects themselves indicated a need for competent pretraining to be able to cope with a social robot [21]. The approach of older people to new technologies, such as assistive robots, depends on many factors, including gender, education level, and previous experience with electronic devices [22,23]. Research to date has revealed that men are more open to new technologies than women [24], and people who have previously used various electronic devices are more likely to get acquainted with emerging technological innovations [19]. Still, it should be kept in mind that the acceptance of robots is a complex process, and these are certainly not the only factors that affect the views and approaches of older people to such devices.

The study by Schwartz et al [25] showed that consumers' decisions regarding the use of services largely depend on the advice given to them by professional experts, such as doctors, financial advisors, and accountants. A series of psychological experiments revealed that people, who have established a long-term relationship with an expert, are quite reluctant to seek additional advice on the services offered to them [26]. The results of this study suggest that the opinions of older people on the use of assistive robots can fundamentally depend on the point of view of health care professionals who take care of them (eg, doctors and nurses). If the attending physicians or formal caregivers of older people (whom they trust and whose knowledge they value) have a positive attitude toward new technologies (eg, assistive robots), it is quite likely that the older people will be more willing to become familiar with such a device and to use it at home. Therefore, it seems important to check what future doctors and nurses think about assistive robots that can support older people.

The aim of this study was to collect the opinions of future health care professionals (medical and nursing students) about the use of robots in the care of older people.

## Methods

### The ENRICHME Project

The study was conducted as part of the ENRICHME (ENabling Robot and Assisted Living Environment for Independent Care and Health Monitoring of the Elderly) project funded by the European Union under the Horizon 2020 framework (project number: 643691), with consent from the Bioethical Committee of the Poznan University of Medical Sciences (consent number: 389/17). The project evaluated the possibility of supporting patients with mild cognitive impairment using an assistive robot in their home environment [27].

### The Studied Group

The study was performed in a group of 178 students from the Poznan University of Medical Sciences, Poznan, Poland (110 nursing students and 68 medical students). The study included only students who completed the second year of study (ie, those who can be reasonably expected to have knowledge about the care of older people). None of the participants indicated previous experience with robotics.

### The Users’ Needs, Requirements, and Abilities Questionnaire

The research was conducted using the Users’ Needs, Requirements, and Abilities Questionnaire (UNRAQ) developed jointly by ENRICHME project partners [21,28]. The UNRAQ is composed of three main parts. The first of these involves demographic data, with questions about age, gender, education, and computer skills. The second part involves opinions about robots (ie, use of robots, roles of robots, social aspects, and assistant role). For each statement in this part of the questionnaire, the respondent had the opportunity to choose the most appropriate answer on a 5-point Likert scale (1, I totally disagree; 2, I partially disagree; 3, I neither agree nor disagree; 4, I partially agree; 5, I totally disagree). Answers 4 and 5 were treated as positive, answer 3 was treated as neutral, and answers 1 and 2 were treated as negative. The third part of the questionnaire involves the so-called creativity box (ie, a place where respondents can present their ideas about robots and their functions). Prior to the interview, the participants were presented with a photograph of the Kompaï robot (Robosoft).

### Statistical Analysis

Data are presented as mean (SD). Statistical calculations were performed using the Statistica 13 software (StatSoft). The Mann-Whitney *U* test was used to assess the statistical significance of differences between the studied groups, where a *P* value <.05 was considered statistically significant.

## Results

### Characteristics of the Studied Groups

The mean age of the participants was 21.6 (SD 2.4) years among nursing students and 23.2 (SD 2.0) years among medical students. All study participants declared that they were able to operate a computer, and the vast majority of them claimed that they were at ease in using electronic devices (148/170, 87.1%; 96 nursing students and 52 medical students). Out of 178 respondents, 23 looked after a family member (15 nursing students and eight medical students).

### Opinions of Students About the Readiness of Older People for Operating a Robot

**Figure 1:**
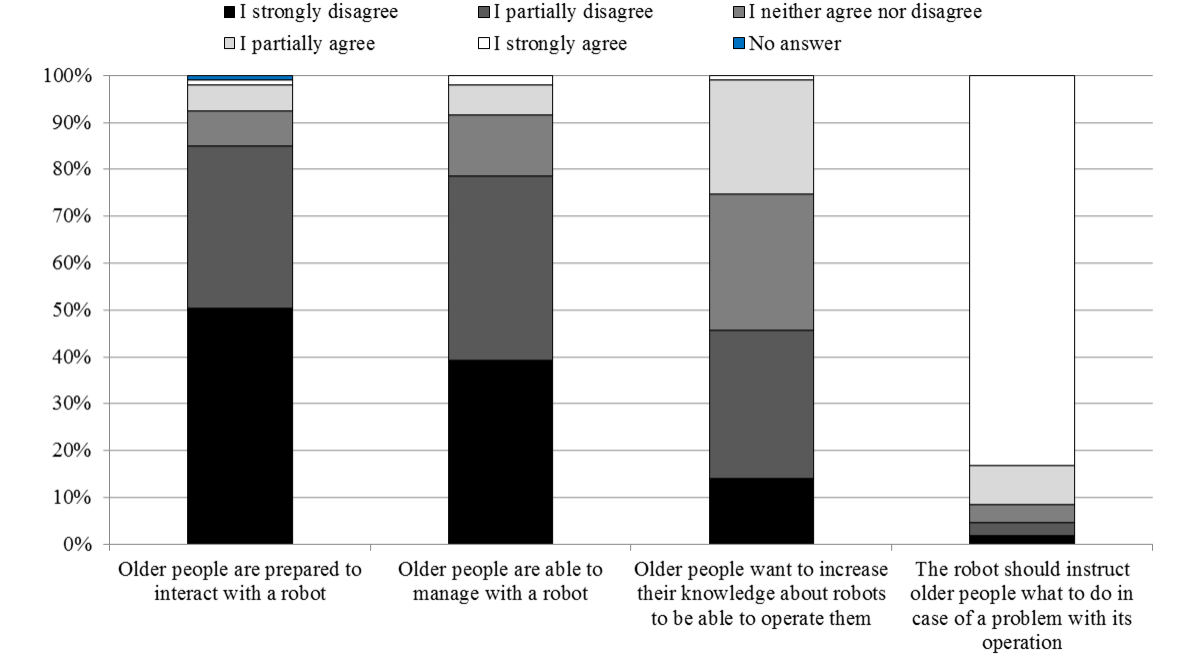
Opinions of the whole group of students about the readiness of older people to use a robot.

The majority of students (151/176, 85.8%) participating in the study thought that older people are currently not prepared for the use of assistive robots. Among these students, 90 strongly agreed with this statement and 61 partially agreed (Figure 1). Only 10% (7/68) of medical students positively assessed the readiness of older people to use a robot. Notably, this opinion was shared by only one nursing student (1/108, 0.9%). The differences observed between the groups were not relevant. The surveyed students believed that older people would like to broaden their knowledge about robots in order to be able to use them, and 30.9% (21/68) of medical students and 17.4% (19/109) of nursing students agreed with this statement (*P*=.07). Respondents in their statements emphasized that there are no forms of education in this field for older adults in Poland and that not all older people can use electronic devices; therefore, robot training should be conducted. The vast majority of students believed that an assistive robot should give the user instructions on its use (164/177, 92.7%).

### Students’ Opinions About the Role of a Robot

A great majority of nursing and medical students (167/178, 93.8%) considered a robot as a useful device for older people. Additionally, majority of students (152/178, 85.4%) considered it as an assistant for older people. Only about 55% of students (96/175, 54.9%) considered a robot as a companion for older people (Table 1). Respondents unanimously emphasized that a robot will not replace relationships with real people and that for older people, it should be only an assistant and not a companion.

**Table 1:** Opinions of nursing and medical students about the general role of a robot in the care of older people.

Statement	Total (N=178)		Nursing students (N=110)		Medical students (N=68)		*P* value
	n^a^ (%^b^)	Mean (SD)	n^a^ (%^b^)	Mean (SD)	n^a^ (%^b^)	Mean (SD)	
A robot is a useful device for older people (something to be used when needed, with no other interaction)^c^	167 (93.8)	4.6 (0.8)	105 (95.5)	4.6 (0.7)	62 (91.2)	4.6 (0.9)	.25
A robot is an assistant for older people	152 (85.4)	4.2 (0.9)	92 (83.6)	4.2 (1.0)	60 (88.2)	4.3 (0.9)	.30
A robot is a companion for older people	96 (54.9)	3.5 (1.2)	59 (54.6)	3.5 (1.2)	37 (55.2)	3.4 (1.2)	.53

^a^Number of students who agreed with the statement (strongly or partially).

^b^Percentage of students who agreed with the statement (strongly or partially).

^c^Data computed for 175 students owing to missing data in three questionnaires.

The answers regarding the use of a robot and its specific tasks in the care of older people were grouped according to the degree of their acceptance by students participating in the study (Table 2). Nine perfect match statements (over 90% positive answers) defined the most important use of an assistive robot concerning mainly functions, such as reminding about medications, ensuring security at home (calling for help if needed), monitoring the user’s health status and the environment, preventing memory deterioration (eg, through cognitive training), and encouraging the user to maintain physical activity. A very good match (71%-90% positive answers) was obtained for 11 statements related, among others, to monitoring the amount of food and fluid intake, as well as providing entertainment to the older user. A good match (51%-70% positive answers) was observed for seven statements, including encouraging users to contact other people and the ability to customize the robot’s functions to the individual needs of the user. However, students emphasized that older people should not be allowed to change the robot’s settings themselves, as this may result in the needed functions being turned off.

**Table 2:** Opinions of nursing and medical students related to statements about the specific functions of a robot in the care of older people, arranged by the percentage of students who agreed with the statement.

Group, Statement		Total (N=178)		Nursing students (N=110)		Medical students (N=68)		*P* value
		n^a^ (%^b^)	Mean (SD)	n^a^ (%^b^)	Mean (SD)	n^a^ (%^b^)	Mean (SD)	
**Perfect match**								
	The robot reminds about medications.	169 (94.9)	4.7 (0.6)	102 (92.7)	4.7 (0.6)	67 (98.5)	4.7 (0.6)	.52
	The robot helps to preserve memory function (eg, plays memory games).	167 (94.9)	4.6 (0.7)	105 (96.3)	4.6 (0.6)	62 (92.5)	4.4 (0.9)	.13
	The robot encourages and guides the owner to perform physical exercises.	167 (93.8)	4.5 (0.7)	100 (90.9)	4.6 (0.7)	67 (98.5)	4.5 (0.6)	.08
	The robot increases the safety of the person: calls for help when needed and monitors health parameters (blood pressure, heart rate, body temperature, respiration rate, etc).	166 (93.3)	4.6 (0.8)	103 (93.6)	4.6 (0.8)	63 (92.6)	4.6 (0.8)	.85
	The robot increases the safety of the home (eg, locks doors, detects leaking gas, etc).	165 (92.7)	4.6 (0.8)	101 (91.8)	4.6 (0.8)	64 (94.1)	4.6 (0.7)	.99
	The robot reminds about meal times and drink times.	165 (92.7)	4.5 (0.8)	102 (93.6)	4.6 (0.7)	62 (91.2)	4.4 (0.9)	.03
	The robot reminds about appointments.	163 (91.6)	4.5 (0.7)	99 (90.0)	4.5 (0.7)	64 (94.1)	4.4 (0.8)	.23
	The robot monitors the environment (temperature and humidity) and suggests air conditioning adjustment or opening windows.^c^	162 (91.5)	4.5 (0.8)	102 (93.6)	4.6 (0.8)	60 (88.2)	4.3 (0.9)	.001
	The robot should be customizable (adjustable to individual user preferences and needs).^c^	160 (90.4)	4.6 (0.8)	96 (88.1)	4.5 (0.8)	64 (94.1)	4.6 (0.8)	.52
**Very good match**								
	The robot provides advice about a healthy diet.	160 (89.9)	4.5 (0.8)	98 (89.1)	4.5 (0.8)	62 (91.2)	4.4 (0.8)	.04
	The robot observes the behavior of the older person to detect falls or changes due to illness.	158 (88.8)	4.5 (0.8)	97 (88.2)	4.5 (0.7)	61 (89.7)	4.5 (0.9)	.96
	The robot monitors the amount of food and fluid intake of the owner.	155 (87.1)	4.3 (0.9)	99 (90.0)	4.4 (0.8)	56 (82.4)	4.1 (1.0)	.02
	The robot informs a family member or caregiver about the older person’s behavior/health problem.	152 (85.4)	4.3 (0.9)	92 (83.6)	4.4 (0.9)	60 (88.2)	4.2 (0.8)	.01
	The robot helps the owner to find lost objects (eg, glasses and keys).	150 (84.3)	4.4 (0.8)	91 (83.6)	4.4 (0.8)	58 (85.3)	4.3 (0.8)	.23
	The robot has entertainment functions (eg, gaming partner, reading aloud, and playing music).	147 (82.6)	4.2 (0.9)	91 (82.7)	4.2 (0.9)	56 (82.4)	4.2 (0.8)	.69
	The robot is able to make the life of older people easier.	146 (82.0)	4.2 (0.8)	88 (80.0)	4.2 (0.8)	58 (85.3)	4.3 (0.8)	.37
	The older person has control over the robot.^c^	134 (75.7)	4.1 (0.9)	79 (72.5)	4.1 (0.9)	55 (80.9)	4.2 (0.9)	.30
	The robot can automatically reactivate after being switched off.	131 (73.6)	4.0 (1.2)	84 (76.4)	4.1 (1.2)	47 (69.1)	3.8 (1.1)	.02
	The older person is able to choose the required functions of the robot and disable other functions.	126 (70.8)	3.9 (1.0)	78 (70.9)	3.9 (1.0)	48 (70.6)	4.0 (1.0)	.31
	The robot has much information about the user (social, medical, and others).^d^	126 (71.6)	3.9 (0.9)	75 (69.4)	3.9 (0.9)	51 (75.0)	3.9 (1.0)	.89
**Good match**								
	The robot initiates contact with others (eg, calls friends and initiates skype conversations).	123 (69.1)	4.0 (1.0)	87 (70.9)	4.0 (1.0)	45 (66.2)	3.9 (1.1)	.35
	The robot encourages to enhance contact with friends.	123 (69.1)	4.0 (1.0)	83 (75.5)	4.1 (0.9)	40 (58.8)	3.7 (1.0)	.003
	The robot accompanies the owner in everyday activities (eg, watching TV and preparing meals).	123 (69.1)	3.8 (1.1)	76 (69.1)	3.9 (1.2)	47 (69.1)	3.8 (1.0)	.32
	The older person is able to send the robot to its place/docking station and keep it staying there.	121 (68.4)	3.9 (1.1)	69 (63.3)	3.8 (1.1)	52 (76.5)	4.0 (1.0)	.10
	The older person is able to switch off the robot in specific situations (friends’ visits, privacy reasons, etc).^c^	121 (68.0)	3.9 (1.2)	68 (61.8)	3.7 (1.2)	53 (77.9)	4.2 (1.0)	.01
	The robot detects the owner’s mood (facial expression).	121 (68.0)	3.9 (1.1)	74 (67.3)	3.9 (1.1)	47 (69.1)	3.8 (1.1)	.54
	The robot decreases the sense of loneliness and improves the mood of the older person.	118 (66.3)	3.7 (1.2)	76 (69.1)	3.9 (1.1)	42 (61.8)	3.4 (1.2)	.008

^a^Number of students who agreed with the statement (strongly or partially).

^b^Percentage of students who agreed with the statement (strongly or partially).

^c^Data computed for 177 students owing to missing data in one questionnaire.

^d^Data computed for 176 students owing to missing data in two questionnaires.

Nursing students scored significantly higher than medical students in the case of eight statements concerning everyday use of robots (reminding about meals [*P*=.03], monitoring the environment [*P*=.001], providing advice about a healthy diet [*P*=.04], monitoring the intake of food and fluids [*P*=.02], and automatic “switch on” function [*P*=.02]). Nursing students were more focused on the social functions of robots (encouraging to enhance contact with friends and reducing the sense of loneliness and improving mood). On the other hand, medical students were more aware of privacy issues in the statement concerning the possibility of switching off the robot in specific situations.

## Discussion

### The Attitude of Future Medical Professionals to Assistive Robots

Over the past several years, we have been experiencing the rapid development of technology that penetrates almost all aspects of life (including the care of older adults). Assistive robots can not only help older people remain independent for longer, but also support and facilitate the work of doctors and formal caregivers [29]. Our research concentrated on the approach of future health care professionals to the use of robots in the care of older people and what roles they think such devices should play. In general, the results of our analyses indicate a positive attitude of medical and nursing students to socially assistive robots. The vast majority of participants saw high potential in such devices, similar to the findings in the study by van Kemenade et al [30] for companion robots. These results are also consistent with the results obtained in focus group studies of potential end users, and younger and older caregivers of older people as part of the Domeo project [31], as well as surveys conducted by Faucounau et al [32] and Cylkowska-Nowak et al [21].

However, it should be emphasized that the surveyed students had some reservations about the use of robots by older people. They suggested that older people in Poland might not yet be ready to use such devices owing to difficulties in handling these devices. In addition, the problem may concern not only the operation of the robots but also the selection of the most suitable model or the setting of functions appropriate for a given user. According to the students, older people often do not have sufficient knowledge of the use of electronic devices or their suitability for potential users’ needs and requirements. The participants of our study pointed to the necessity to provide specific training to older people on the use of robots. This corresponds with the observation of Flandorfer that the more experienced people are in using new technologies and the smarter the devices, the higher is the desire to use such devices when needed [18]. Johansson-Pajala et al also observed that the attitudes of study participants to care robots improved as their knowledge increased [33], which was interpreted as a general need for an improved orientation within the field.

### The Role of Robots According to Future Medical Professionals

Our study revealed that the most important roles of assistive robots relate to functions such as reminding about taking medications, ensuring the safety of older people, preventing deterioration of their memory, and encouraging them to maintain physical activity. Future doctors and nurses were most critical of using a robot as a companion of an older person. Although the results of previous studies indicated that older people can benefit from such interactions [34], students involved in our research believed that robots should never replace contact with other people, but rather should encourage such contact.

The study participants also believed that assistive robots should be personalized (ie, it should be possible to select appropriate functions in relation to the individual needs of a given user, depending, among others, on the state of health). Notably, surveyed students believed that older people should not be able to change the robot settings themselves. They argued that an older person could disable the functions that are most needed (eg, those for monitoring their health and detecting a threat), thereby possibly exposing themselves to danger.

### Differences in Opinions Among Future Medical Staff About Assistive Robots

We noticed slight differences between the studied groups. Nursing students were more open to robots and saw more opportunities for their use in the care of older people compared with medical students. This is reflected in their higher average results regarding positive responses and acceptance of a larger spectrum of robot functions (very good and perfect match). They were also more aware of the problems related to the aging process, such as restricted social life, which can cause negative social effects, as well as undernourishment or drinking too little fluid, which may have health consequences (eg, lead to cognitive decline) [35].

### The Worries of Future Medical Staff About Assistive Robots

Although the development of robotics may raise some ethical issues [36], none of the students participating in our study showed any ethical concerns about who could decide to turn off the robot or set it up, similar to the results in other studies [31]. The surveyed students believed that older people should not be able to turn off the robot completely for their safety.

However, it should be kept in mind that, when creating such devices, it is necessary to not only improve the lives of older people by enabling them to live in their homes longer, but also protect their individual rights and their physical and mental well-being [37,38].

Among future medical staff, we did not observe a fear of losing their jobs from the deployment of assistive robots. The students very clearly emphasized in their answers that robots will never replace contact with another person (social issues). These devices should only act as an assistant to formal caregivers and doctors, helping them to care for older people and monitor their health remotely. Importantly, students showed concerns about older caretakers, indicating the possibility of an emotional bond with the device and the negative consequences it could have in the event of damage or failure of the robot.

### Doctors and Nurses as Professional Experts

The study by Schwartz et al [25] proved that the opinions and approaches of people to use certain services might be influenced by professional advisers, such as doctors and nurses. This effect is particularly evident in the case of a long-term relationship between the expert and the client, as well as when the client values the expert’s knowledge and experience. Therefore, the positive attitude of future medical personnel to assistive robots observed in our study can presumably have an impact on the acceptance of robots by older people. It is also worth noting that doctors and nurses as formal caregivers could additionally facilitate the introduction of robots into the homes of their patients. The research conducted so far proves that it is easier for younger generations to use new technologies with which they have grown and that it is easier for them to make a more accurate choice of the right device [39]. Therefore, medical staff (from younger generations than their patients) could certainly help older people in choosing devices that would best suit their needs. Importantly, as doctors and nurses know the needs of older people, they should actively cooperate with engineers at as early a stage as possible in robot technology design. Such collaboration allows designing better robots, which could consequently maximize the independence of older people while reducing health care costs. The study by Hoenig et al [40] showed that people who needed assistance in their daily activities and who did not use assistive devices needed an average of 4 hours a week of additional professional care compared with people who used such devices. This seems particularly important if we take into account the fact that the world population is aging, and thus, fewer people can act as formal caregivers.

### Assistive Robots: Changes in the Training of Medical Staff

Our study findings indicate that future doctors and nurses are profoundly interested in the functioning of older adults and are happy to comment on the possibilities of using assistive technologies. However, it appears that changes in the curriculum of courses aimed at gerontechnology are desirable, and such classes would be particularly important in terms of doctors and nurses acting as experts or advisers for older people and their cooperation with designers on the necessary functions and features of assistive robots. Skiba [41] emphasized that lecturers for nursing students should introduce gerontechnology to the curriculum to reflect important trends that occur in the society (technology development), and this is especially so, as older people are willing to learn how to use new technologies to be able to stay at home longer, which is extremely important for them [6,42]. Following the suggestion of van Kemenade et al, there should be a place to address the ethical concerns related to the use of robots in care [30]. Curricula at medical universities should include basic engineering concepts as well, which will allow doctors and nurses to evaluate and select the right device (thus helping clients) and will facilitate the solution of possible handling problems arising during its use [43]. As the implementation of socially assistive robots in care requires a partnership among academic institutions, clinicians, and the industry [30], interprofessional collaboration involving highly skilled nurses and medical doctors is indispensable.

### Limitations of Our Study and Future Research Directions

We are aware that the relatively small number of participating students is a limitation of our research. In addition, the students did not have the opportunity to observe the use of an assistive robot in the care of older people in practice, which could have affected the results of the study. Broadbent et al [44] found that attitudes toward a robot improve after interacting with it. This suggests that conducting such research after giving the participants an opportunity to use an assistive robot in practice could result in even higher acceptance by future doctors and nurses. In future studies, it would be worth investigating to what extent such interaction with a robot increases its acceptance. Additionally, differences in the attitudes of students from different countries and regions (eg, Japan vs Europe) are worth exploring [45]. Moreover, the inclusion of potential end users from older generations as well as other stakeholders (family members, professional caregivers, etc) might yield noteworthy results, as indicated in a range of previous studies [20,28,31,33].

### Conclusions

Our research revealed a generally positive attitude of future doctors and nurses toward assistive robots. According to the participants of this study, an assistive robot should primarily remind older people to take medications regularly, ensure their safety, monitor their health status and environment, counteract the deterioration of their memory, and encourage them to maintain physical activity. In the opinion of the students, such a robot should not be an older person’s companion but should rather act as an assistant. The positive attitude of future medical personnel (professional experts) toward assistive robots, as demonstrated by us, can have a relevant impact on the acceptance of such devices by older adults. In the future, doctors and formal caregivers could help their patients choose the right robots (and the necessary functions) that are best suited to their needs. However, this would require expanding the scope of university education to include questions on gerontechnology.

## Abbreviations

ENRICHME: ENabling Robot and Assisted Living Environment for Independent Care and Health Monitoring of the Elderly
UNRAQ: Users’ Needs, Requirements, and Abilities Questionnaire
